# Cardiac magnetic resonance follow-up of COVID-19 vaccine associated acute myocarditis

**DOI:** 10.3389/fcvm.2022.1049256

**Published:** 2022-11-09

**Authors:** Dmitrij Kravchenko, Alexander Isaak, Narine Mesropyan, Leon M. Bischoff, Claus C. Pieper, Ulrike Attenberger, Daniel Kuetting, Sebastian Zimmer, Christopher Hart, Julian A. Luetkens

**Affiliations:** ^1^Department of Diagnostic and Interventional Radiology, University Hospital Bonn, Bonn, Germany; ^2^Quantitative Imaging Lab Bonn, University Hospital Bonn, Bonn, Germany; ^3^Department of Internal Medicine II–Cardiology, University Hospital Bonn, Bonn, Germany; ^4^Department of Pediatric Cardiology, University Hospital Bonn, Bonn, Germany

**Keywords:** cardiac magnetic resonance, myocarditis, COVID-19, vaccination, follow-up

## Abstract

**Background:**

Mass COVID-19 vaccination campaigns have helped impede the COVID-19 pandemic. In rare cases, some vaccines have led to vaccine associated myocarditis in a specific subset of the population, usually young males. Cardiac magnetic resonance (CMR) can reliably diagnose vaccine associated myocarditis, but follow-up data of CMR proven acute myocarditis is scarce.

**Materials and methods:**

Nine patients with acute vaccine associated myocarditis underwent baseline and follow-up CMR examinations and were compared to baseline parameters at initial presentation and to a group of 20 healthy controls. CMR protocol included functional assessment, T1 and T2 mapping, T2 signal intensity ratio, strain feature tracking, and late gadolinium enhancement (LGE).

**Results:**

Myocarditis patients (*n* = 9, aged 24 ± 6 years, 8 males) underwent CMR follow-up after an average of 5.8 ± 4.3 months. All patients showed a complete resolution of visual myocardial edema while also demonstrating a reduction in overall LGE extent from baseline to follow-up (4.2 ± 2.1 vs. 0.9 ± 0.8%, *p* < 0.001), although visual LGE was still noted in all patients. Left ventricular ejection fraction was normal at baseline and at follow-up (58 ± 6 vs. 62 ± 4%, *p* = 0.10) as well as compared to a healthy control group (60 ± 4%, *p* = 0.24). T1 (1024 ± 77 vs. 971 ± 34 ms, *p* = 0.05) and T2 relaxations times (57 ± 6 vs. 51 ± 3 ms, *p* = 0.03) normalized at follow-up. Most patients reported a resolution of clinical symptoms, while two (22%) reported new onset of exertional dyspnea.

**Conclusion:**

Patients with COVID-19 vaccine associated acute myocarditis showed a complete, uncomplicated resolution of myocardial inflammation on follow-up CMR, which was associated with a near complete resolution of symptoms. Minor, residual myocardial scarring was present on follow-up LGE imaging. The long-term implications of the remaining myocardial scar-tissue after vaccine associated myocarditis remain unknown warranting further studies.

## Introduction

The start of the coronavirus disease-19 (COVID-19) outbreak caused by the severe acute respiratory syndrome coronavirus 2 (SARS-CoV-2) at the end of 2019 prompted a world-wide effort to curtail the spread of the pandemic. One possible way to achieve this was the development of safe and effective vaccines. This effort cumulated in a rapid deployment of a few COVID-19 vaccines, most commonly and notably the Ad26.COV2-S [recombinant] (Janssen) vaccine, the mRNA-1273 (Moderna) vaccine, the BNT162b2 (Pfizer/BioNTech) vaccine, and the ChAdOx1-S [recombinant] (AstraZeneca) vaccine. Shortly after mass immunization programs began, reports of vaccine associated adverse reactions, such as fever, deep venous thrombosis, and myocarditis, started to emerge. Myocarditis and pericarditis are rare cardiovascular adverse vaccine reactions with an estimated incidence of approximately 0.48 cases per 100,000 administered vaccines (1). The Vaccine Adverse Event Reporting System (VAERS), set up by the Disease Control and Prevention (CDC) and the U.S. Food and Drug Administration (FDA), currently record 94 cases of reported vaccine associated myocarditis for the Janssen vaccine, 980 for the Moderna vaccine and 1897 for the BioNTech vaccine (2). Typical clinical presentation includes new exertional dyspnea and/or acute chest pain, accompanied by elevated troponin T, hours to days after COVID-19 vaccination. Cardiac magnetic resonance (CMR) studies have shown that the pattern of myocardial involvement in vaccine associated myocarditis was similar to acute viral induced myocarditis (3–5). In accordance with the 2018 modified Lake Louise Criteria for the diagnosis of acute myocarditis, typical findings include generalized or focal edema, prolonged T1 or T2 relaxation times, increased extracellular volume (ECV), and focal necrosis on late gadolinium enhancement (LGE) imaging (6). Initial clinical studies suggest a mild clinical course of disease with rapid resolution of symptoms (7). However, there is currently a scarcity of follow-up studies, making it difficult to determine the possible risks associated with vaccine associated myocarditis. This study reports follow-up CMR findings in patients after initial acute vaccine associated myocarditis.

## Materials and methods

This retrospective study was approved by the appropriate institutional ethics committee and performed in concordance with the Declaration of Helsinki and International Conference on Harmonization of Good Clinical Practice. The requirement for written informed consent was waived. Patients who underwent initial CMR with diagnosis of acute vaccine-associated myocarditis and subsequent follow-up CMR at the Department of Diagnostic and Interventional Radiology from September 2021 to August 2022 were retrospectively identified.

Initial symptoms for referral for CMR included exertional dyspnea, chest pain, fever, or palpitations with associated elevated troponin T, within hours or days of receiving a COVID-19 vaccine. All patients received at least one dose of a COVID-19 vaccine approved for use in the European Union. Initial CMR results were positive for myocarditis as defined by the 2018 Lake Louise criteria (6). Reasons for follow-up referrals were standardized CMR follow-up of acute myocarditis according to local guidelines, examination before return to physical activity, or persistent cardiac symptoms under exertion. Clinical patient information was gathered through the local hospital information system.

The control group consisted of healthy subjects without previous myocarditis and no cardiovascular disease history who underwent CMR for study control reasons. Controls were age-matched to the myocarditis cohort and had normal CMR results without structural abnormalities.

### Cardiac magnetic resonance protocol

All CMR examinations were performed using clinical whole-body MRI systems (Ingenia 1.5T or 3.0T; Philips Healthcare, Best, The Netherlands). Signal reception was achieved by a 16-channel torso coil using a digital interface. A signal intensity correction algorithm (CLEAR: Constant LEvel AppeaRance; Philips Medical Systems) was utilized to correct for torso-coil related signal inhomogeneities. Short-axis, 2-chamber, 3-chamber, and 4-chamber cine views were acquired using electrocardiogram gated, breath-hold steady state free precession sequences for functional analysis. A transversal respiratory-gated fat-suppressed T2-weighted fast spin echo sequence (Philips MultiVane XD, Philips Healthcare, Best, The Netherlands) was acquired for the assessment of axillary and mediastinal lymphadenopathy, which was also included in the follow-up protocol. Myocardial edema was visualized using T2-weighted short-tau inversion-recovery sequences in short axis and transversal views. T2 STIR images were also used to calculate T2 signal intensity ratio. Myocardial T1 and T2 mapping was performed in end-diastolic short axis views with acquisition of apical, midventricular, and basal sections. A six-echo gradient spin-echo sequence (GraSE) was applied for myocardial T2 mapping (8). Myocardial T1 mapping was achieved using a standard 3(3)3(3)5 modified Look-Locker inversion recovery (MOLLI) acquisition scheme, with post-contrast T1 maps acquired 10 min after the administration of contrast medium (9). For contrast enhancement, a 0.2 mmol/kg of body weight bolus of gadoterate meglumine (Clariscan; GE Healthcare, Chicago, IL, USA) was used. Segmented inversion-recovery gradient-echo sequences for LGE imaging were obtained in short axis, 2-chamber, 4-chamber, and transversal views. The Look-Locker method was utilized to determine the optimal inversion time for LGE image acquisition as previously described (10). Sequence parameters for 1.5 Tesla are summarized in Supplementary Table 1.

### Image analysis

Image analysis was performed by a board-certified cardiovascular radiologist (J.A.L with 10 years of experience in CMR) and a radiology resident (D.K. with 4 years of experience in CMR) using dedicated software (IntelliSpace Portal, version 12.1.4.; Philips Medical Systems). Papillary muscles were included for the volumetric quantification of the left ventricle. Global systolic radial, longitudinal, and circumferential strain were calculated by using feature tracking strain analysis software (CAAS MR Solutions, version 5.2.1.; Philips Medical Systems) using short-axis, 2-chamber, and 4-chamber balanced steady state free precession cine imaging.

Focal areas of regional high signal intensities in a non-ischemic distribution pattern on T2 short-tau inversion-recovery and on LGE images were visually assessed by consensus agreement of the two readers. Quantitative markers of myocardial edema (T2 signal intensity ratio) and myocardial injury and fibrosis (enhanced areas were defined as those with a signal intensity ≥ 3.0 standard deviations above the mean signal intensity of normal myocardium) were calculated as previously reported (11–13). Motion correction was achieved using a software-implemented algorithm (fast elastic image registration, IntelliSpace Portal) for myocardial T1 and T2 relaxation maps, deriving global T1 and T2 relaxation times. Hematocrit-corrected global ECV values were calculated as previously described (12, 14, 15). For scans at 1.5 Tesla, institution specific cutoffs (≥1000 ms for myocardial T1 relaxation times and ≥ 55.9 ms for myocardial T2 relaxation times) for the assessment of the 2018 Lake Louise criteria were used as previously described (16). LGE distribution was classified according to the American Heart Association 17 segment heart model (17). LGE localization was classified according to wall involvement (subepicardial, midmyocardial, subendocardial, transmural, or patchy). Axial T2 weighted images were assessed for axillary lymph node enlargement and compared to previous imaging in the injection arm of the vaccine. For the control group, the largest axillary lymph node of either side was used. The largest short axis diameter measured in millimeter was recorded.

### Statistical analysis

Prism (version 8.4.1; GraphPad Software) and Jamovi (version 2.2; The Jamovi Project) were used for statistical analysis. Data are given as means ± standard deviation or as percent to absolute frequency. Continuous variables were summarized as median with interquartile range (IQR) or as mean ± standard deviation, as appropriate. Normal distribution was checked using the Shapiro–Wilk test. For comparison of continuous variables and inter-individual variables the Student’s *T*-test was used. A paired *T*-Test was used for the comparison of means in variables recorded at baseline and at follow-up. Mann–Whitney-*U* test was used for non-normal distributed data. Dichotomous variables were compared by using the χ2 test. One-way analysis of variance (ANOVA) followed by Tukey *post-hoc* multiple comparison tests was performed to compare variables in three groups. The level of statistical significance was set to *P* < 0.05.

## Results

### Patient characteristics

In total, nine patient datasets with baseline and follow-up CMR (8 male [89%], aged 24 ± 6 years) were available for retrospective analysis. A detailed comparison with 20 age-/and gender-matched controls (18 males [89%], aged 26 ± 7 years) is given in Table 1. Patients with vaccine associated myocarditis received the following vaccines: Pfizer/BioNTech (*n* = 7, 78%) of which one was a first dose, four were the second dose, and two were a booster dose (third vaccination); Moderna (*n* = 1, 11%) of which it was a second dose; Janssen (*n* = 1, 11%) first dose (only one dose required). Highly sensitive troponin T levels were elevated in all nine patients (median 644 ng/l [IQR: 159–930 ng/l]). All patients were treated with either cardioprotective or anticoagulative medication: 6 out of 9 (67%) were treated with beta-blockers, 4 (44%) with ACE-inhibitors, 2 (22%) with low molecular weight heparin, and one (11%) with a diuretic.

**TABLE 1:** Patient characteristics and cardiac magnetic resonance findings.

Parameter	Acute myocarditis baseline (*n* = 9)	Acute myocarditis follow-up (*n* = 9)	Controls (*n* = 20)	*P*-value
Age (years)	24.1 ± 6.4	24.7 ± 6.1	25.9 ± 7.2	0.77
Males (n, %)	8 (89)	8 (89)	18 (89)	0.99
Height (cm)	176 ± 9	176 ± 9	177 ± 8	0.98
Weight (kg)	74 ± 20	78 ± 22	79 ± 11	0.84
LVEF (%)	58 ± 6	62 ± 4	60 ± 4	0.29
LVEDV (ml)	158 ± 32	161 ± 28	157 ± 20	0.95
LVEDVi (ml/m^2^)	83 ± 12	82 ± 12	80 ± 8	0.62
IVSD (mm)	8.7 ± 1.8	9.2 ± 1.6	9.3 ± 1.7	0.69
Visual edema (n)^b^	7 (77%)	0 (0%)	0 (0%)	<0.001*
Visual LGE present (n)^b^	9 (100%)	9 (100%)	0 (0%)	<0.001^†^
LGE extent (%)	4.2 ± 2.1	0.9 ± 0.8	0.6 ± 0.2	<0.001*
T2 signal intensity ratio	1.9 ± 0.3	1.7 ± 0.3	1.6 ± 0.3	0.07
T1 relaxation times (ms)^a^	1024 ± 77	971 ± 34	982 ± 62	0.28
T2 relaxation times (ms)^a^	57 ± 6	51 ± 3	51 ± 3	0.07
ECV (%)	24.5 ± 2.1	24.3 ± 1.8	23.3 ± 1.9	0.29
GRS (%)	25.6 ± 6.4	30.2 ± 10.2	22.9 ± 4.8	0.13
GCS (%)	−12.7 ± 1.9	−14.9 ± 2.7	−13.5 ± 2.0	0.17
GLS (%)	−17.0 ± 2.2	−16.4 ± 1.9	−16.2 ± 1.7	0.60
Largest axillary lymph node at injection side (mm)	11.8 ± 2.3	7.8 ± 2.3	6.4 ± 1.3	<0.001*

*P*-values were derived from ANOVA (with Tukey *post-hoc* tests) unless otherwise noted. ^a^Data from one patient was not included due to CMR examination at 3.0 Tesla. ^b^χ2 test. *p < 0.05 baseline compared to follow-up and control. ^†^*p* < 0.05 control group compared to baseline and follow-up. IQR, interquartile range; LVEF, left ventricular ejection fraction; LVEDV, left ventricular end diastolic volume; LVEDVi, left ventricular end diastolic volume index; IVSD, interventricular septum thickness at diastole; LGE, late gadolinium enhancement; ECV, extra cellular volume; GRS, global radial strain; GLS, global longitudinal strain; GCS, global circumferential strain.

### Clinical symptoms

All nine had clinical symptoms of acute myocarditis at initial scan: 8 out of 9 (89%) presented with chest pain, 3 (33%) with exertional dyspnea, and 2 (22%) with occasional fever. At follow up, only 1 out of 9 (11%) patients reported persistent chest pain, 2 patients reported new onset of exertional dyspnea (22%), and none reported fever. Patients did not report signs of infection prior to vaccination. Median number of days to symptom onset after vaccination was 0 days (IQR: 0–1 days; mean 0.6 ± 1.0 days), median time to initial CMR was 6.5 days (IQR: 5.3–12.8 days; mean 8.1 ± 3.9 days), and median time to follow-up was 3.0 months (IQR: 2.0–10.5; mean 5.8 ± 4.3 months).

### Cardiac magnetic resonance results

**FIGURE 1:**
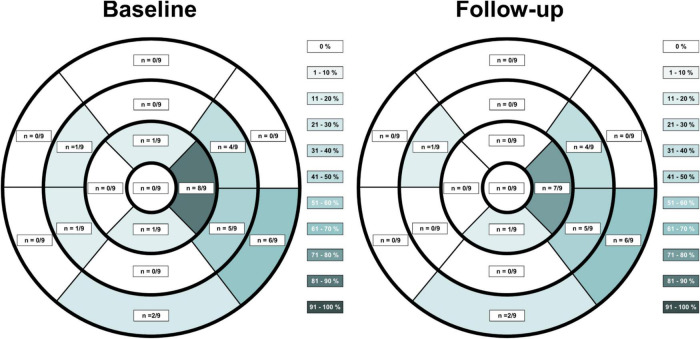
Late gadolinium enhancement (LGE) distribution on CMR in patients suffering from vaccine associated myocarditis at acute baseline imaging and at follow-up according to the American Heart Association 17-segment heart model. LGE extent was reduced on follow-up imaging, although LGE findings were still discernable at most previous locations.

**FIGURE 2:**
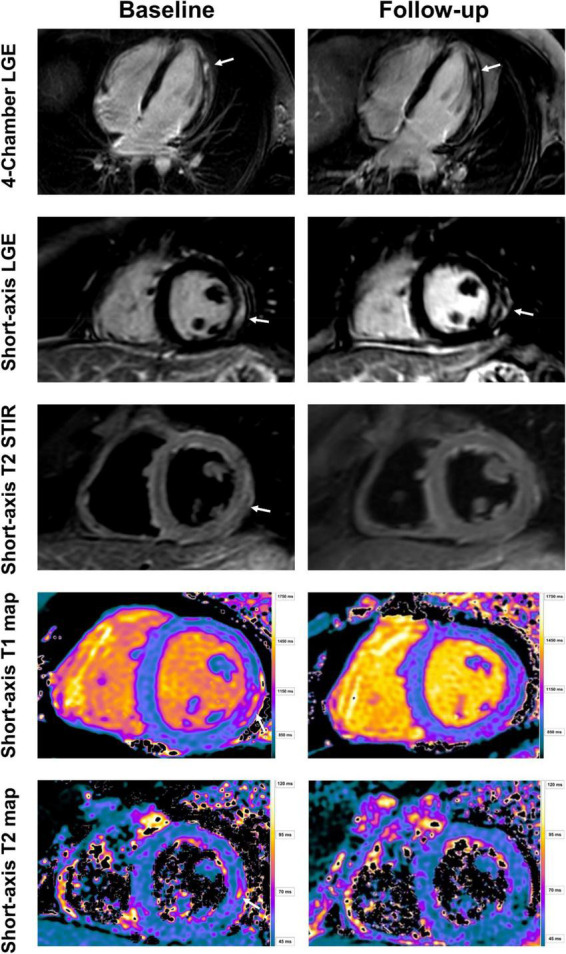
Baseline and follow-up cardiac imaging in a 25-year-old male with acute vaccine associated myocarditis after receiving his second vaccine with Pfizer/BioNTech. Short-axis and 4-chamber late gadolinium enhancement (LGE) views demonstrate subepicardial enhancement along the midventricular and apical inferolateral wall (arrows). T2 short-axis short tau inversion recovery (STIR) imaging corresponding to the LGE findings shows a resolution of edema from baseline to follow-up (arrow). Normalization of T1 and T2 relaxation times are also demonstrated over time (arrow). Note, however, the persistent LGE along the inferolateral wall even at follow-up, consistent with scar tissue.

**FIGURE 3:**
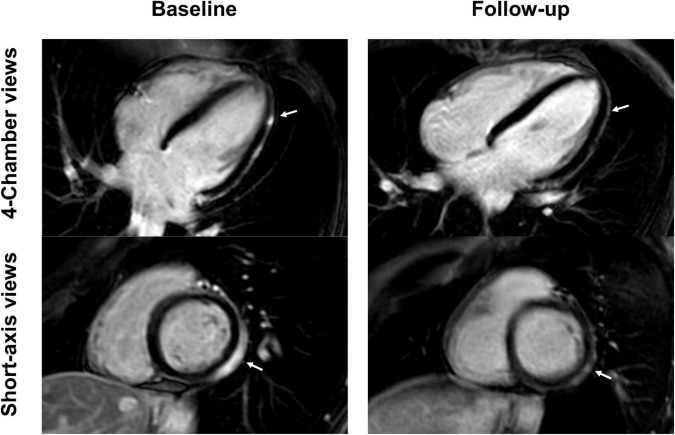
Short-axis and 4-chamber late gadolinium enhancement (LGE) views in a 28-year-old, previously healthy male after receiving his second Moderna vaccine. Reduction of subepicardial enhancement along the lateral wall from baseline (arrows) to follow-up 11-months later consistent with myocardial scarring. This patient reported new onset of occasional exertional dyspnea after initial acute vaccine associated myocarditis.

**FIGURE 4:**
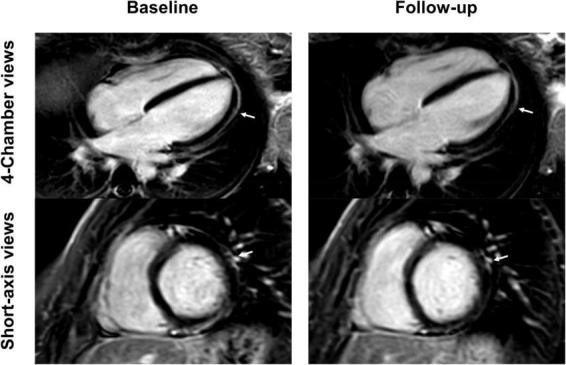
Short-axis and 4-chamber late gadolinium enhancement (LGE) views in a 26-year-old, previously healthy female after receiving the first dose of the Pfizer/BioNTech vaccine. Nearly complete resolution of LGE (arrows) at follow-up 2 months later with minor enhancement discernable at the apical lateral segment. Clinical correlation showed a complete resolution of previous symptoms which included chest pain and exertional dyspnea.

**FIGURE 5:**
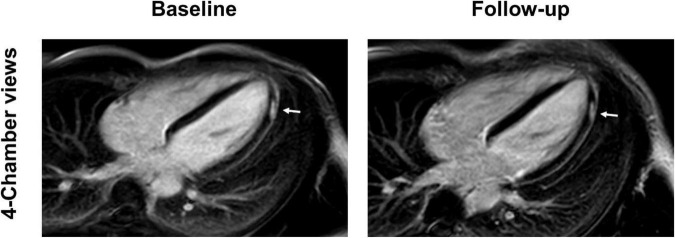
4-chamber late gadolinium enhancement (LGE) views of a 36-year-old, previously healthy male after receiving the third dose of the Pfizer/BioNTech vaccine. The LGE lesion at the apical lateral wall (arrow) reduced at follow-up 3 months later, but was still visible, a finding which is consistent with scar tissue. Patient reported complete resolution of clinical symptoms which included chest pain and palpitations.

**FIGURE 6:**
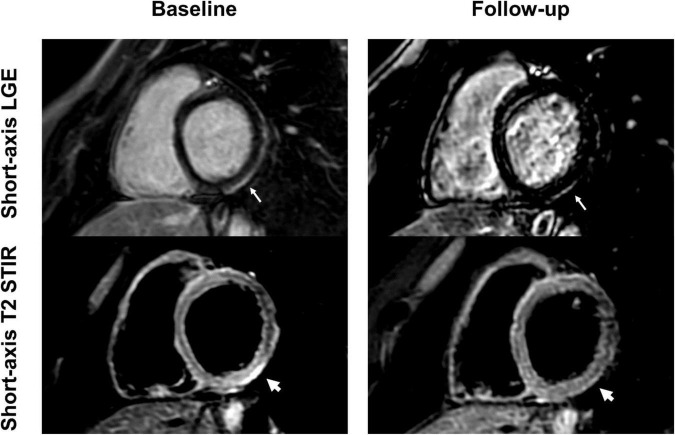
Short-axis late gadolinium enhancement (LGE) and short-axis T2 short tau inversion recovery (STIR) views of a 24-year-old, previously healthy male after receiving the second dose of the Pfizer/BioNTech vaccine. LGE at the basal inferolateral wall (thin arrows) shows a marked decrease at 6-month follow-up, with only minimal remaining findings. T2 STIR imaging shows focal myocardial edema corresponding to the location of the LGE (thick arrows) with complete resolution at follow-up. The patient reported complete resolution of previous clinical symptoms but complained of new onset exertional dyspnea.

**FIGURE 7:**
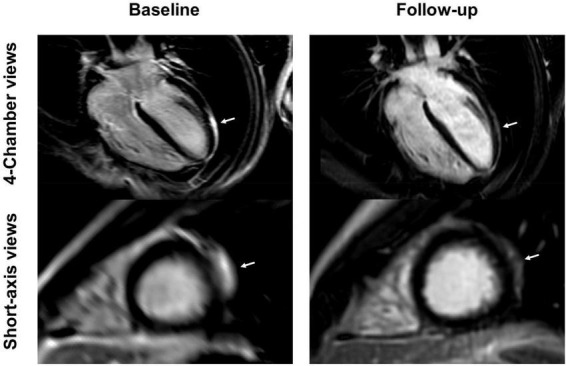
4-chamber and short-axis late gadolinium enhancement (LGE) views of a 15-year-old male after receiving the second dose of the Pfizer/BioNTech vaccine. Subepicardial and pericardial LGE at the apical lateral wall (arrows) shows a marked decrease at the 9-month follow-up with residual findings. Resolution of chest pain at follow-up was accompanied by new onset of occasional exertional dyspnea.

T1 and T2 relaxation times from the same scanner at the same field strength (1.5T) were available for 8 patients while one patient received both, baseline and follow-up scans using a 3.0T MRI scanner. T1 and T2 maps from this patient were excluded from consecutive analysis. All patients demonstrated positive LGE findings typical for acute myocarditis. The most common LGE distribution pattern was subepicardial (*n* = 9, 100%), followed by midwall (*n* = 3, 33%) or diffuse transmural involvement (*n* = 2, 22%). LGE distribution according to the American Heart Association 17-segment heart model at baseline and at follow-up is depicted in Figure 1. Visual myocardial edema was noted in 7 out of 9 patients at baseline (78%) with complete resolution at follow-up in all patients. Direct baseline to follow-up imaging comparisons are shown in Figures 2–7. All *p*-values within the text are derived from a paired *t*-test analysis between baseline and follow-up data unless otherwise noted. All patients demonstrated a significant reduction of overall LGE extent from baseline to follow-up (4.2 ± 2.1 vs. 0.9 ± 0.8%, *p* = 0.001), although persistent LGE was noted in all patients at follow-up, consistent with post-inflammatory scar tissue. In two cases (22%) visual LGE on follow-up was barely discernable, and in all cases visual LGE demonstrated a reduction in signal intensity. Septal LGE sparring was noted in most patients and only 2 patients demonstrated septal LGE. T2 relaxation times were noted to be significantly higher at baseline compared to follow-up (57 ± 6 vs. 51 ± 3 ms, *p* = 0.03). T1 relaxation times were also higher at baseline, although not statistically significant (1024 ± 77 vs. 971 ± 34 ms, *p* = 0.05).

No significant differences between baseline and follow-up investigation were noted for left ventricular ejection fraction (58 ± 6 vs. 62 ± 4%; *p* = 0.10), left ventricular end diastolic volume (158 ± 32 vs. 161 ± 28 ml; *p* = 0.62), or left ventricular end diastolic volume index (83 ± 12 vs. 83 ± 9 ml/m^2^; *p* = 0.87). A significant reduction in axillary lymph node size was noted between baseline and follow-up (11.8 ± 2.3 vs. 7.8 ± 2.3 mm; *p* = 0.003). A slightly significant improvement in systolic global circumferential strain (GCS) was noted from baseline to follow-up (−12.7 ± 1.9 vs. −14.9 ± 2.7%, *p* = 0.04). No such statistically significant difference was noted for systolic global radial strain (GRS, 25.6 ± 6.4 vs. 30.2 ± 10.2%, *p* = 0.12) or global longitudinal strain (GLS, −17.0 ± 2.2 vs. −16.4 ± 1.9%, *p* = 0.08) between baseline and follow-up. Table 2 provides an overview of some currently available data regarding vaccine associated myocarditis.

**TABLE 2:** An overview of current studies concerning COVID-19 vaccine associated myocarditis.

References	Patients (n)	Males (n)	Age (years)	1st/2nd dose (n)	Vaccine P/M/A/J (n)	Symptoms	CMR findings
Ahmed et al. (18)	7	7	25	0/7	5/2/0/0	Chest pain, fatigue, dyspnoea, elevated troponin T	Acute non-severe myocarditis after vaccination
Evertz et al. (19)	10	10	26	2/8	4/6/0/0	Chest pain, dyspnoea	Subepicardial LGE and edema, normal LVEF, normal global longitudinal strain
Fronza et al. (4)	21	17	31	4/17	9/12/0/0	Chest pain	LGE findings in all patients
Kravchenko et al. (3)	9	7	24	2/7	8/1/0/0	Chest pain, elevated troponin T, fatigue	All LLC positive patients demonstrated elevated troponin T and LGE on CMR
Abellan et al. (20)	3	3	29	0/3	0/3/0/0	Chest pain, elevated troponin T	Acute non-severe myocarditis after vaccination
Diaz et al. (21)	20	15	36*	4/16	9/11/0/0	N/A	Acute non-severe myocarditis or perimyocarditis after vaccination
Bautista García et al. (22)	1	1	39	0/1	1/0/0/0	Fever, chest pain	Edema and LGE
Isaak et al. (23)	1	1	15	0/1	1/0/0/0	Fever, myalgia, chest pain, elevated troponin T	Subepicardial LGE. Normal left ventricular function
Jain et al. (24)	63	58	16	1/62	59/4/0/0	Fever, chest pain, fatigue, headache	Mild LVEF dysfunction, edema, LGE
Kim et al. (25)	4	3	38	0/4	2/2/0/0	Fatigue, chest pain	All patients demonstrated subepicardial LGE and elevated T1 and T2 times
Larson et al. (26)	8	8	32	1/7	5/3/0/0	Chest pain	Elevated troponin T in 6 patients. All patients demonstrated LGE findings, most with associated oedema
Marshall et al. (27)	7	7	17*	0/7	7/0/0/0	Chest pain, elevated troponin T	All patients presented with LGE, hyperaemia, and cardiac oedema
Montgomery et al. (28)	23	23	25*	3/20	7/16/0/0	Chest pain, elevated troponin T	CMR was performed in 8 of 23 cases with findings including edema and abnormal LGE
Abu Mouch et al. (29)	6	6	–	1/5	5/0/0/0	Chest pain	Elevated troponin T in 4 out of 6. All patients demonstrated LGE. Uncomplicated resolution
Perez et al. (30)	7	6	50	1/6	3/4/0/0	Chest pain, dyspnoea, fatigue	LGE, pericardial involvement in 50% of the cases
Rosner et al. (31)	7	7	27	2/7	5/1/0/1	Chest pain, elevated troponin T, fever	Cardiac edema in 5 patients. LGE in all patients
Shaw et al. (32)	4	2	24	2/2	3/1/0/0	Chest pain	Edema and LGE
Truong et al. (7)	139	126	16	12/128	131/5/0/1	Chest pain, fever, myalgia	Edema and LGE

P, Pfizer/BioNTech; M, Moderna; A, AstraZeneca; J, Johnson & Johnson; N/A, not available; CMR, cardiac magnetic resonance; LGE, late gadolinium enhancement; LLC, Lake Louise criteria; LVEF, left ventricular ejection fraction. All data are presented as mean unless otherwise noted. *Data reported as median.

## Discussion

This retrospective study analyzed clinical and CMR data from nine, non-hospitalized patients with vaccine associated myocarditis regarding parameters such as left ventricular function, LGE extent, and myocardial T1 and T2, at initial imaging and at follow-up as well as to an age and sex matched control group. While vaccine associated myocarditis is a rare possible complication of currently available COVID-19 vaccines, its clinical presentation and CMR characteristics at the acute stage and follow-up should be known to cardiovascular imaging physicians.

Vaccine associated myocarditis is more likely to occur after vaccination with mRNA-based vaccines, although we report of one case after vaccination with a vector vaccine (Janssen). The number of received doses also seems to play a role as vaccine associated myocarditis is less likely to be noted after the first dose, usually causing symptoms after the second or third booster dose, suggesting that prior exposure is necessary for the development of vaccine associated myocarditis. However, the exact pathomechanism of vaccine associated myocarditis remains unknown. Current theories focus on a hypersensitivity reaction or cross reactions of spike proteins with myocardial contractile proteins (33, 34).

Collaborating previously published findings, we found vaccine associated myocarditis to predominantly affect younger males (1, 3, 35). Typically, clinical course of disease is mild with rapid resolution of symptoms within a few months (17, 19, 25, 28, 33), although persistent LGE on CMR indicative of fibrous scar tissue was noted in all our patients. Resolution of other CMR findings (elevated T1 and T2 times, focal or diffuse edema) are to be expected as the extent of the inflammatory process diminishes over time. Other studies have noted a relatively normal left ventricular ejection fraction in vaccine associated myocarditis compared to patients with other causes of myocarditis (4). We found similar findings in our study, as parameters of myocardial function were not noticeably impaired. Cardiac strain has been shown to improve the diagnostic performance of the 2018 revised Lake Louise Criteria and provide prognostic value regarding major adverse cardiovascular events for acute myocarditis (36–39). Strain analysis might be able to detect subtle changes in myocardial tissue earlier when other traditional prognostic markers such as left ventricular ejection fraction (LVEF) or LGE are normal. Strain encoded MRI has been reported to be able to identify patients with subclinical LVEF dysfunction, potentially at risk for heart failure (40). In ischemic heart disease, strain encoded MRI was able to differentiate between reversible and irreversible myocardial injury (41). Reduced GLS and GCS have also been associated with edema in suspected acute myocarditis (42). Feature tracking strain analysis did not show any statistically significant difference in systolic GRS or GLS between the three groups. A decrease in GLS has been previously described to be a negative prognostic marker for major adverse cardiovascular events (36). A small, but discernable improvement was noted for GCS going from baseline to follow-up, as well as an insignificant improvement in GRS and worsening in GLS. The difference in these values might be attributed to the small patient population of this study. Resolution of symptoms was noted in most patients, although two patients reported new onset of occasional exertional dyspnea. This may be in part due to residual LGE findings on CMR, which has been previously described as a marker of unfavorable prognosis when paired with a resolution of associated edema (43). The full implications of remaining myocardial scar tissue are unknown in such a young patient group. The risk of developing ventricular arrhythmias for such young patients after vaccine-induced acute myocarditis is currently unknown. A preference for lateral ventricular wall involvement for LGE with septal sparring has been observed in the majority of patients, indicating a more favorable prognosis regarding the development of arrhythmias (44–47).

Our study suffers from limitations, including the small patient cohort and variable follow-up times. Most statistical comparisons were not corrected for multiple testing due to the small data set. As of date, there are no published consensus criteria for the diagnosis of vaccine associated myocarditis after COVID-19 vaccination. We therefore defined it as myocarditis like symptoms after COVID-19 vaccination with abnormal CMR findings in previously healthy people accompanied with elevated troponin T levels within a reasonable number of days of COVID-19 vaccination. Not all CMR examinations were performed on the same machine or at the same field strength, diminishing data sets available for statistical analysis. Furthermore, the reference standard for diagnosis of myocarditis, endomyocardial biopsy, was not performed as it is not part of the best standard of care practice at our institution. Further studies comparing CMR findings in vaccine associated myocarditis with other causes of myocarditis akin to data published by Fronza et al. offer an outlook for potential future research (35).

## Conclusion

Vaccine associated myocarditis tends to affect younger, predominantly male patients and shows abnormal CMR findings such as focal or diffuse edema, elevated T1 and T2 relaxation times, and LGE. While the overall prognosis seems to be favorable and a rapid resolution of symptoms is observed, reduced, yet persistent LGE findings indicative of myocardial fibrosis in light of complete resolution of edema have been noted. Further studies are needed to examine the long-term effects of the remaining scar-tissue and develop recommendations for patients with a history of vaccine associated myocarditis regarding booster doses.

## Data availability statement

The raw data supporting the conclusions of this article can be provided upon reasonable request by the authors, without reservation.

## Ethics statement

The studies involving human participants were reviewed and approved by Ethikkommission der Medizinischen Fakultät Bonn, University Hospital Bonn, Venusberg-Campus 1, 53127, Bonn. Written informed consent from the participants or their legal guardian/next of kin was not required to participate in this study in accordance with the national legislation and the institutional requirements.

## Author contributions

JL and DKr contributed to the conception and design of the study and performed the statistical analysis. DKr organized the database and wrote the first draft of the manuscript. JL, AI, DKu, DKr, CP, and SZ wrote sections of the manuscript. All authors contributed to the manuscript revision, read, and approved the submitted version.
