# Supplementary material for: Cardiac magnetic resonance follow-up of COVID-19 vaccine associated acute myocarditis
Source: Front Cardiovasc Med. 2022 Nov 9;9:1049256. doi: 10.3389/fcvm.2022.1049256 (PMC9682292; doi:10.3389/fcvm.2022.1049256)
Supplement: Supplementary file 1 [file Table_1.docx]

**Table A1. MRI sequence parameters for 1.5 Tesla field strength magnets.**

| **Parameter** | **Short axis SSFP cine** | **Short axis black-blood T2 STIR** | **Short axis LGE** | **T1 Mapping** | **T2 Mapping** | **T2 MultiVane XD** |
| --- | --- | --- | --- | --- | --- | --- |
| Field of view (mm) | 352 x 352 | 352 x 352 | 360 x 311 | 300 x 300 | 300 x 347 | 400 x 400 |
| Time of repetition (ms) Time to echo (ms) | 2.8 1.38 | 2 RR intervals 70 | 3.5 1.71 | 2.2 1.02 | 1 RR interval 23.6/ ∆TE = 11.8 (6Ec) | 1114 60 |
| Flip angle (°) | 60 | 90 | 15 | 35 | 90 | 90 |
| Voxel size (mm^3^) acquired reconstructed | 1.79 x 2 x 8 0.99 x 0.99 x 8 | 1.51 x 2.43 x 8 0.91 x 0.91 x 8 | 1.65 x 1.88 x 10 0.9 x 0.9 x 5 | 1.97 x 2 x 10 1.17 x 1.17 x 10 | 1.97 x 2.03 x 10 1.03 x 1.03 x 10 | 1.49 × 1.49 × 5  0.83 × 0.83 × 5 |
| Parallel imaging factor | 3 | 2.5 | 2 | 2 | 2 | 1.5 |
| Scan duration | 1 min 24 sec | 01 min 36 sec | 27 sec | 45 sec | 42 sec | 3 min 54 sec |
| Scan time/ breath-hold (s) | 00:13 | 00:08 | 00:12 | 00:15 | 00:14 | - |
| Cardiac phases per RR interval Shot duration (ms) | 40  - | -  134 | -  151 | -  167 | 83 | -  - |

SSFP, steady-state free precession; STIR, short-tau inversion recovery; LGE, late gadolinium enhancement; Ec, Echo.
